# Gene signature-based mapping of immunological systems and diseases

**DOI:** 10.1186/s12859-016-1012-y

**Published:** 2016-04-18

**Authors:** Hong Liu, Jessica Liu, Michelle Toups, Timothy Soos, Christopher Arendt

**Affiliations:** Bio-Innovation, Sanofi Global Biotherapeutics, 38 Sidney Street, Cambridge, MA 02139 USA; Bioinformatics Program, Northeastern University, 360 Huntington Avenue, Boston, MA 02115 USA

**Keywords:** Gene signature, Gene set, Immune disease, Immune cell, Enrichment analysis, Network-based analysis

## Abstract

**Background:**

The immune system is multifaceted, structured by diverse components that interconnect using multilayered dynamic cellular processes. Genomic technologies provide a means for investigating, at the molecular level, the adaptations of the immune system in host defense and its dysregulation in pathological conditions. A critical aspect of intersecting and investigating complex datasets is determining how to best integrate genomic data from diverse platforms and heterogeneous sample populations to capture immunological signatures in health and disease.

**Result:**

We focus on gene signatures, representing highly enriched genes of immune cell subsets from both diseased and healthy tissues. From these, we construct a series of biomaps that illustrate the molecular linkages between cell subsets from different lineages, the connectivity between different immunological diseases, and the enrichment of cell subset signatures in diseased tissues. Finally, we overlay the downstream genes of drug targets with disease gene signatures to display the potential therapeutic applications for these approaches.

**Conclusion:**

An *in silico* approach has been developed to characterize immune cell subsets and diseases based on the gene signatures that most differentiate them from other biological states. This modular ‘biomap’ reveals the linkages between different diseases and immune subtypes, and provides evidence for the presence of specific immunocyte subsets in mixed tissues. The over-represented genes in disease signatures of interest can be further investigated for their functions in both host defense and disease.

**Electronic supplementary material:**

The online version of this article (doi:10.1186/s12859-016-1012-y) contains supplementary material, which is available to authorized users.

## Background

The immune system has evolved to confer effective host defense in diverse environmental conditions, but it can also be diverted to mediate inflammatory diseases when the system is dysregulated [1]. The complexity of this system is reflected in the multiple immunocyte subsets that co-regulate each other and perform distinct functions at different developmental states, in various tissue microenvironments, and in response to different stimuli [1]. Advances in genomics technology have facilitated the generation of large-scale data sets, including many that provide open access. A major challenge is how to leverage informatics approaches to achieve integrative analyses of multi-scale genomics data in the synthesis of meaningful biological hypotheses and insights [2].

Gene signatures are gene sets that are defined as groups of genes linked by biological relationships that could reflect their common downstream biological programs or functions, as well as their co-regulation based on common inductive networks or chromosomal locations [3]. In the present study, we focus on gene sets that are coordinately regulated under specific biological conditions or across multiple biological states. In contrast to conventional analysis approaches, the use of gene set improves tolerance to non-specific noise and variability between samples, batches, or platforms, and can lead to novel interpretations of large-scale genomic data.

One purpose of developing gene signatures is for the use of GSEA (Gene Set Enrichment Analysis), which evaluates ranked gene lists from genomic profiles for identifying statistically enriched gene sets with defined biological annotation [3]. The Molecular Signatures Database (MSigDB) from the Broad Institute was developed for this purpose, and now contains thousands of gene sets that were analyzed from transcriptional profiles [4]. While single-gene analysis finds little similarity across independent studies; GSEA reveals many biological pathways in common [3]. Chaussabel et al. have focused on co-clustered gene sets, also called modules, in the mining and interpretation of large-scale genomic data through a reductionist approach. They have shown that the use of coordinately expressed gene sets (modules) improves robustness when comparing results across platforms and studies [5, 6].

In an effort to distinguish specific inflammatory mechanisms that are unique or common among different chronic inflammatory or autoimmune diseases, we applied a gene signature approach to develop an integrative immunogenetic biomap. First, we selected from GEO and ArrayExpress databases the genomic studies documenting immune cell lineages, as well as inflammatory or autoimmune disease states. We then enriched for transcriptional signatures associated with each of the cell subtypes and disease states. By clustering and integrating these gene signatures, we uncovered novel connections between diverse inflammatory and autoimmune states and revealed common nodal points for potential therapeutic intervention.

## Results

### Immune cell type gene signatures

**Fig. 1:**
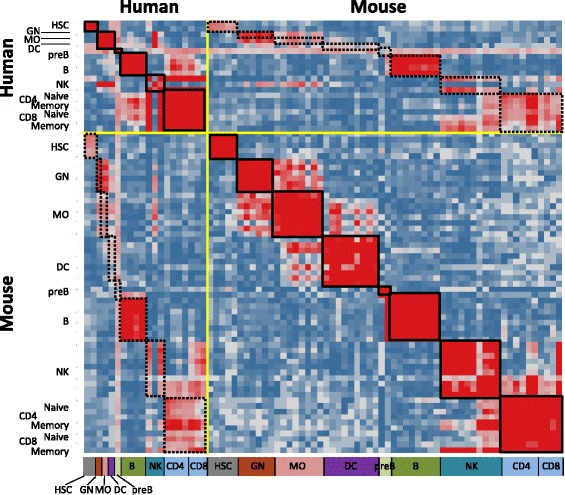
Similarity matrix of immune cell type gene signatures from human and mouse. Seventy-eight immune cell type gene signatures (20 human and 58 mouse) were paired against each other. Similarity was calculated by Fisher’s exact test of overlapping genes for each pair. Gene signatures were positioned according to their common cell lineage. Color represents the –log (*P* value of Fisher’s exact test), with *red color* indicating high similarity, and *blue color* indicating less/no similarity. *Solid line black boxes* group the gene signatures from the same lineage in either human or mouse, while *dotted line black boxes* group those from the same lineage between human and mouse. *HSC* hematopoietic stem cell, *GN* granulocyte, *MO* monocyte

Shay et al. [7] evaluated the conservation of genome-wide expression profiles of human vs. mouse cell types through correlation analysis, assessing the relatedness of matched lineages across species. A recent study by Godec et al. suggests that the lineage specific differences in human and mouse hematopoietic cells can be recapitulated by gene sets [8]. We sought to extend those approaches to formulate cell lineage- and subtype-specific gene sets that could serve as highly enriched ‘signatures’ in additional multivariate analyses. Two methods were evaluated to select these ‘signature’ gene sets, the first collecting the 2 % of genes with the highest expression values in a given subtype, and the second representing the 2 % of genes with the highest specific expression across all subtypes [9]. Gene sets generated from specific subtypes show high similarity across different cell lineages, but less conservation between human and mouse (data not shown). In contrast, gene sets generated across all subtypes are more conserved between species while showing more restricted similarity within the same cell lineage (Fig. 1 and Additional file 1: Figure S1). Since we were most interested in gene signatures with the capacity to discriminate between immune cell lineages, we focused our subsequent studies on gene sets generated across all subtypes.

We examined the cell surface (CD, cluster of differentiation) molecules and cytokine receptors common among at least half of the gene modules associated with a specific cell lineage. As shown in Table 1, we could identify ‘signature’ molecules of particular lineages, including CD300LB and CD44 in granulocyte; CD300A, IL10RA, CD68, and CX3CR1 in monocyte; CD19, CD37, CD38, CD72, IL21R, and CD79B in B cell; CD2 in T cell; CD74 and XCR1 in dendritic cell; and CD244 in natural killer cell, among others [10]. However, a few of them need to be further investigated for their potential functions in the related cell lineages. For example: CD101 and CD14 in granulocyte; CD55 and CD200 in B cell; and CD97 in NK cell could not be identified by publications as known markers for these cell types.

**Table 1:** Over-represented CD molecules and cytokine receptors in immune cell type gene signatures

Cell type	Symbol	Entrez gene name	# of gene set
HSPC	CD34	CD34 molecule	4 out of 5
GN	CD300LB	CD300 molecule-like family member b	6 out of 6
GN	CD300LF	CD300 molecule-like family member f	6 out of 6
GN	CD33	CD33 molecule	6 out of 6
GN	CXCR2	chemokine (C-X-C motif) receptor 2	6 out of 6
GN	CCR1	chemokine (C-C motif) receptor 1	5 out of 6
GN	CD101	CD101 molecule	5 out of 6
GN	CD300LD	CD300 molecule-like family member d	4 out of 6
GN	CXCR1	chemokine (C-X-C motif) receptor 1	4 out of 6
GN	IFNLR1	interferon, lambda receptor 1	4 out of 6
GN	IL13RA1	interleukin 13 receptor, alpha 1	4 out of 6
GN	CD14	CD14 molecule	3 out of 6
GN	CD44	CD44 molecule (Indian blood group)	3 out of 6
GN	TNFRSF1A	tumor necrosis factor receptor superfamily, member 1A	3 out of 6
MO	CD300A	CD300a molecule	7 out of 8
MO	TNFRSF1B	tumor necrosis factor receptor superfamily, member 1B	7 out of 8
MO	IL10RA	interleukin 10 receptor, alpha	7 out of 8
MO	IL17RA	interleukin 17 receptor A	6 out of 8
MO	TNFRSF1A	tumor necrosis factor receptor superfamily, member 1A	5 out of 8
MO	IL13RA1	interleukin 13 receptor, alpha 1	5 out of 8
MO	CD68	CD68 molecule	5 out of 8
MO	TNFRSF21	tumor necrosis factor receptor superfamily, member 21	5 out of 8
MO	CX3CR1	chemokine (C-X3-C motif) receptor 1	5 out of 8
MO	CD244	CD244 molecule, natural killer cell receptor 2B4	4 out of 8
DC	CD74	CD74 molecule, major histocompatibility complex, class II invariant chain	9 out of 9
DC	IL10RA	interleukin 10 receptor, alpha	8 out of 9
DC	XCR1	chemokine (C motif) receptor	5 out of 9
B	CD19	CD19 molecule	9 out of 10
B	CD37	CD37 molecule	9 out of 10
B	CD38	CD38 molecule	9 out of 10
B	CD79A	CD79a molecule, immunoglobulin-associated alpha	9 out of 10
B	CD79B	CD79b molecule, immunoglobulin-associated beta	9 out of 10
B	CD22	CD22 molecule	8 out of 10
B	CD55	CD55 molecule, decay accelerating factor for complement (Cromer blood group)	8 out of 10
B	CD72	CD72 molecule	8 out of 10
B	CD74	CD74 molecule, major histocompatibility complex, class II invariant chain	8 out of 10
B	CXCR5	chemokine (C-X-C motif) receptor 5	8 out of 10
B	TNFRSF13B	tumor necrosis factor receptor superfamily, member 13B	8 out of 10
B	TNFRSF13C	tumor necrosis factor receptor superfamily, member 13C	8 out of 10
B	CD180	CD180 molecule	7 out of 10
B	CCR6	chemokine (C-C motif) receptor 6	6 out of 10
B	CD200	CD200 molecule	5 out of 10
B	IL21R	interleukin 21 receptor	5 out of 10
B	IL9R	interleukin 9 receptor	5 out of 10
NK	IL2RB	interleukin 2 receptor, beta	10 out of 10
NK	IL12RB2	interleukin 12 receptor, beta 2	9 out of 10
NK	CCR5	chemokine (C-C motif) receptor 5 (gene/pseudogene)	6 out of 10
NK	CD244	CD244 molecule, natural killer cell receptor 2B4	6 out of 10
NK	CD97	CD97 molecule	6 out of 10
NK	IL12RB1	interleukin 12 receptor, beta 1	6 out of 10
NK	IL18RAP	interleukin 18 receptor accessory protein	6 out of 10
NK	CMKLR1	chemokine-like receptor 1	5 out of 10
NK	IL18R1	interleukin 18 receptor 1	5 out of 10
T	IL27RA	interleukin 27 receptor, alpha	9 out of 10
T	CD6	CD6 molecule	8 out of 10
T	CD2	CD2 molecule	7 out of 10
T	CD5	CD5 molecule	7 out of 10
T	IL21R	interleukin 21 receptor	7 out of 10
T	CD4	CD4 molecule	6 out of 10
T	CD28	CD28 molecule	5 out of 10
T	CD3D	CD3d molecule, delta (CD3-TCR complex)	5 out of 10
T	CD3E	CD3e molecule, epsilon (CD3-TCR complex)	5 out of 10
T	TNFRSF25	tumor necrosis factor receptor superfamily, member 25	5 out of 10

### Immune disease gene signatures

In order to understand the connectivity of different immune diseases, we investigated the similarity of dysregulated genes between chronic inflammatory and autoimmune conditions. To accomplish this, we constructed 155 gene signatures derived from independent studies on nine different immune-related diseases (Table 2) that represent collections of genes which are upregulated in disease samples compared to normal controls.

**Table 2:** Immune disease gene signatures

Disease category	# of gene set	# of study	# of signature disease gene
COPD	20	13	169
Asthma	20	10	11
Dermatitis	12	8	51
Psoriasis	7	5	161
IBD	19	8	100
Lupus	25	17	154
Arthritis	20	11	55
Sclerosis	13	7	1
T1D	19	8	15

“# of Gene set” indicates the number of gene sets for each disease category; “# of Study” indicates the number of independent studies that were analyzed to generate the gene sets; “# of Signature Disease Gene” indicates the number of genes existing in more than five gene sets from at least two different studies. *COPD* chronic obstructive pulmonary disease, *IBD* inflammatory bowel disease, *T1D* type 1 diabetes

**Fig. 2:**
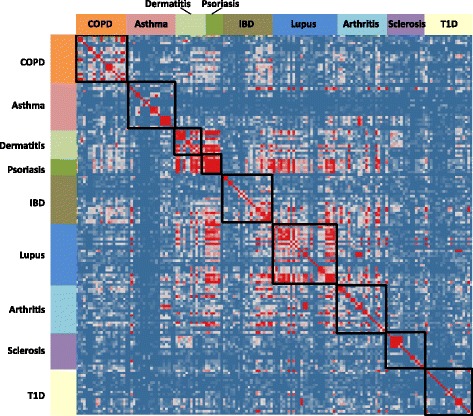
Similarity matrix of immune disease gene signatures. One hundred fifty-five Immune disease gene signatures were paired against each other. Similarity was calculated by Fisher’s exact test of overlapping genes for each pair. Gene signatures from the same disease category were positioned together. *Color* represents the –log (*P* value of Fisher’s exact test), with *red color* indicating high similarity, and *blue color* indicating less/no similarity. *Black boxes* group the gene signatures that represent the same disease category

The similarity matrix derived from these disease gene signatures illustrates that gene signatures from the same disease tend to cluster with one another (Fig. 2). In addition, gene signatures from the same tissue origin, for instance dermatitis and psoriasis, showed higher similarity to each other than to those from other tissues. Most lupus gene signatures were from studies based on blood samples. They show high similarity among themselves, cluster closely with those from synovial fluid (arthritis), and also show cross-similarity to some of the gene signatures generated from colon mucosal biopsies (IBD). In contrast, gene signatures for sclerosis and T1D are distinct from those of other diseases. Those derived from different tissue samples are very different from each other although they represent the same disease (Additional file 1: Figure S2).

We next investigated the over-represented genes in these immune disease signatures. For each disease category, we identified genes common to at least five different gene sets and generated from at least two different studies. We classified these as ‘signature’ disease genes, which are presented in Table 2 for each disease category. Consistent with what we observed from the disease similarity matrix, more ‘signature’ disease genes were found for COPD, psoriasis, lupus, and IBD than for sclerosis, asthma and T1D. Despite the smallest total number of gene sets for psoriasis, the number of signature disease genes is quite large compared to other diseases. This may reflect consistency in disease biology between patients, but could also be due to less heterogeneity at the level of skin tissue samples relative to biopsies involving other tissue types.

**Fig. 3:**
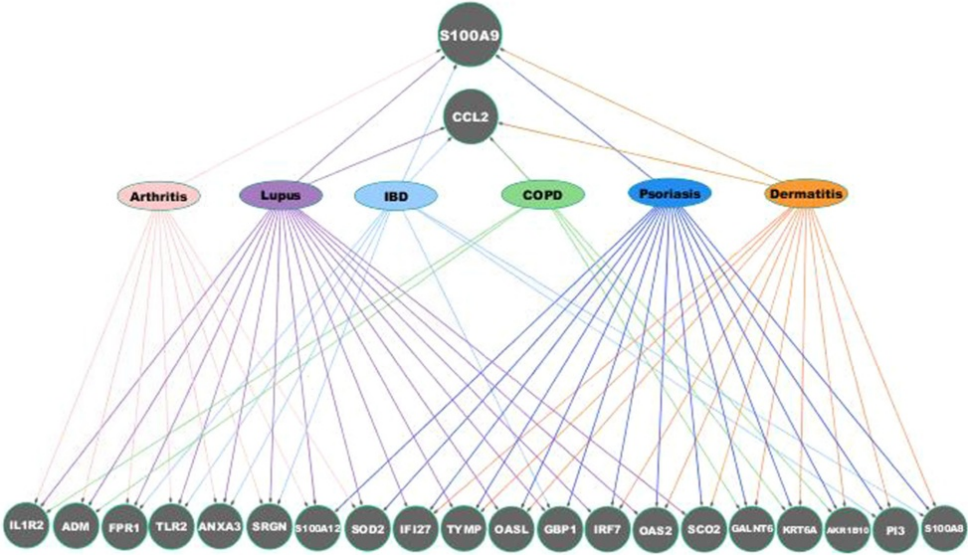
Over-represented genes in immune disease modules. Twenty-two upregulated genes, found to be common to more than three signature disease gene lists, are illustrated. S100A9 is common to 5 diseases while CCL2 is common among 4 different diseases

Figure 3 shows genes common to three or more of the signature disease gene lists. Of particular note, S100A9 is associated with most diseases, including arthritis, lupus, IBD, psoriasis, and dermatitis. This implies that it is up-regulated in a high percentage of samples associated with those diseases. The second most highly represented gene is CCL2, which links with four diseases: lupus, IBD, COPD, and dermatitis. None of the genes in Fig. 3 are common to asthma, sclerosis, or T1D, which is consistent with the smaller overall numbers of associated signature disease genes for these diseases.

**Fig. 4:**
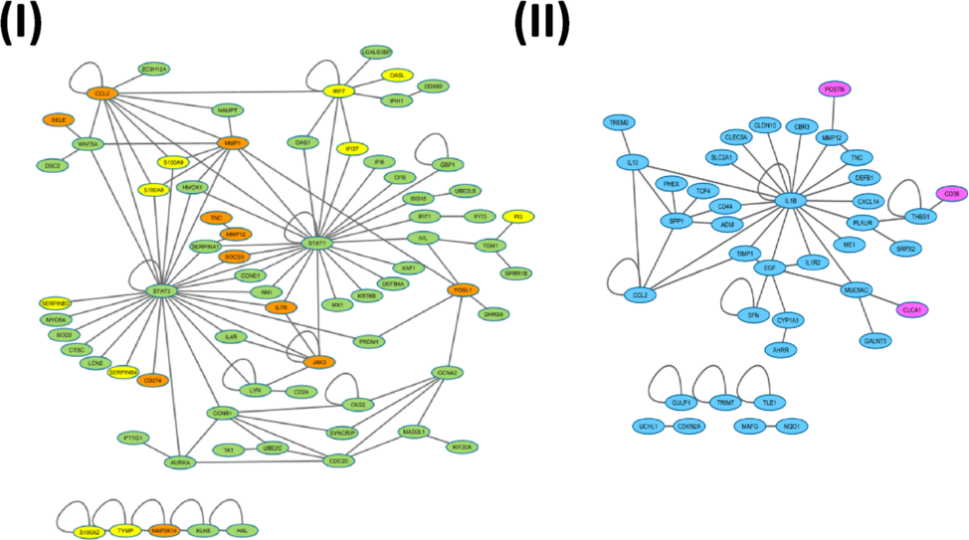
Topological localization of pair of disease genes. Signature disease genes representing two different disease categories and with direct links on the interactome were plotted. (**I**) Topological locations of dermatitis and psoriasis genes. *Orange color*: genes unique to dermatitis; *green color*: genes unique to psoriasis; *yellow color*: genes shared by dermatitis and psoriasis. (**II**) Topological locations of COPD and asthma genes. *Blue color*: genes unique to COPD; *rose color*: genes unique to asthma

**Fig. 5:**
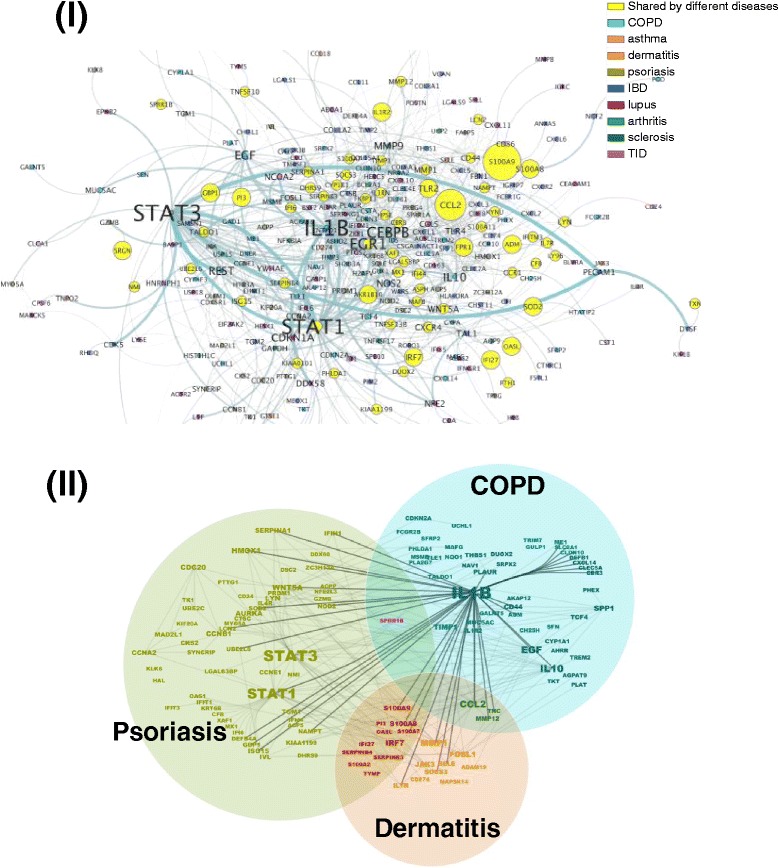
The interactions of signature disease genes. (**I**) Signature disease genes representing all nine disease categories with direct links on the interactome were plotted. The size of the gene name label is proportional to the number of interactions (degrees). Different *colors* represent different disease categories, while *yellow* represents genes shared by multiple diseases. The size of the *yellow circle* is proportional to the number of diseases to which that common gene belongs. (**II**) Signature disease genes representing three disease categories and with direct links on interactome were plotted. The size of the gene name is proportional to the number of interactions. The *thick lines* depict the direct IL1B interactions

To further evaluate the network-based relationships of signature disease genes, we mapped them to an interactome. To reduce noise and avoid over-linkage, only genes with direct links were retained. In Fig. 4, disease genes from dermatitis and psoriasis were revealed to share common genes, as well as linked genes, while COPD and asthma do not share common genes. Furthermore, genes unique to COPD or asthma contain fewer connections, and are distinct from each other. To quantify and assess the network-based separation of disease genes from different disease categories, we performed pair-wise analysis to calculate the network-based separation score [11] for each pair of disease genes. In Table 3, negative scores indicate that disease genes share overlapping ‘neighborhoods’. These results agree with what we observed in Fig. 2. There are more molecular commonalities between dermatitis, psoriasis, lupus, IBD, and possibly arthritis, than that of other diseases studied. We mapped signature disease genes from all disease categories to a single interactome and represented the number of interactions by gene label size, and the number of diseases it belongs to by node size (Fig. 5). We observed that, in Fig. 5I, many genes shared by multiple diseases (shown in yellow) contain more interactions with other genes, such as STAT1, EGR1, TLR2, CCL2, etc. However, it’s worth noting that genes unique to a disease can also hold a lot of interactions with other signature disease genes, such as STAT3, IL1B, CEBPB, IL10, MMP9, TLR4, EGF, etc. In Fig. 5II, we limited the signature disease genes to three indications: psoriasis, dermatitis, and COPD, which clearly shows their interactions across indications. For example, IL1B, a gene target of multiple drugs targeting different autoimmune diseases, is a signature disease gene for COPD. However, it does not only interact with multiple signature disease genes of COPD, but also directly interacts with many signature disease genes of psoriasis and dermatitis.

**Table 3:**
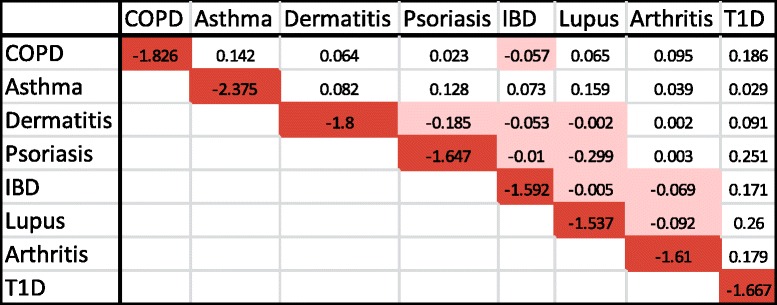
Network-based separation of signature disease genes

Network-based separation analysis was used to calculate the separation score for each pair of signature disease genes. Red color highlights the separation score of a pair with itself.  Pink color highlights the negative separation score.

With the availability of disease gene signatures, we further evaluated the expression level changes of genetically linked genes identified by GWAS (Genome Wide Association Studies). In order to do so, we extracted genetically linked immune disease genes reported in the GWAS catalog [12], and examined their presence in the related disease gene sets. Table 4 shows the number of common genes between GWAS identified genes and genes that are included in disease gene signatures. However, except for psoriasis, the overlap is not significant for most of the disease categories.

**Table 4:** Immune disease gene signatures and genetic linked genes

Disease category	# of GWAS gene	# of DEG	# of overlapped gene
COPD	113	3137	11
Asthma	195	1783	13
Dermatitis	53	2314	5
Psoriasis	56	1144	9*
IBD	525	4095	116
Lupus	181	3696	33
Arthritis	301	5227	83
Sclerosis	343	2041	28
T1D	166	3144	20

“# of GWAS Gene” indicates the number of genes reported in GWAS Catalog that are linked to the related disease category; “# of DEG” indicates the number of Differentially Expressed Genes present in any related disease gene sets; “# of Overlapped Gene” indicates the number of genes existing in both “GWAS Gene” and “DEG”. * indicates that the overlapping is significant based on Chi-square test

### Immune cell type signatures vs. Immune disease signatures

**Fig. 6:**
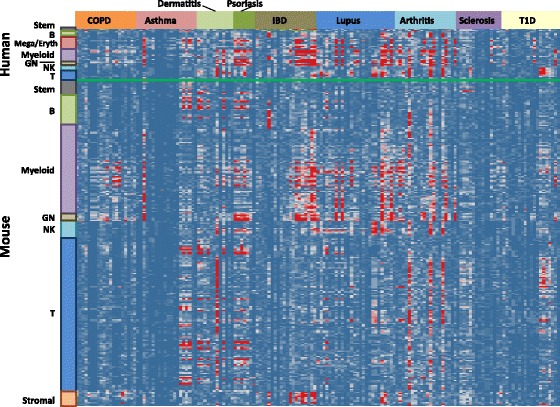
Similarity matrix of immune cell type signatures vs. immune disease signatures. Two-hundred-eighty-seven human and mouse immune cell type signatures were paired against 155 immune disease signatures. Similarity was calculated by Fisher’s exact test of overlapping genes for each pair. Cell type signatures were positioned according to their common cell lineage, and disease signatures were positioned according to their disease category. *Color* represents the –log (*P* value of Fisher’s exact test), with *red color* indicating high similarity, and *blue color* indicating less/no similarity

Autoimmune diseases involve immune cell activation and recruitment to the disease tissue. With the availability of both immune cell type signatures and disease signatures, we evaluated their similarity, for the purpose of elucidating the enrichment of cell type signatures in disease tissue. Among all the cell type signatures, some from the myeloid lineage show the most enrichment with different diseases (Fig. 6). In addition, subsets of cell type signatures are more enriched a subpopulation of disease signatures (Additional file 1: Figure S3). For example, some signatures from either T cell or B cell lineages show enrichment in signatures obtained from dermatitis, psoriasis, asthma and arthritis. Some signatures from the myeloid lineage are enriched in IBD and certain populations of lupus. Stromal modules, however, are enriched mainly in IBD signatures. This finding supports previous observation [13].

### Immune drug target gene sets vs. Immune disease gene signatures

**Fig. 7:**
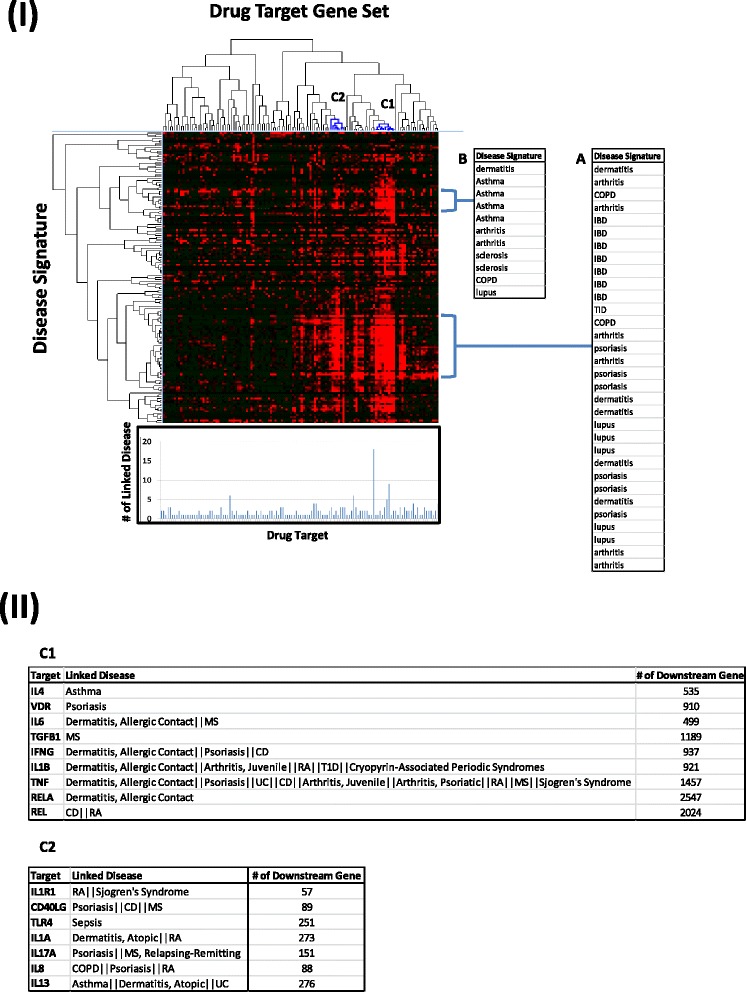
Clustering of immune drug target gene sets vs. immune disease signatures. One-hundred-twenty-six immune drug target gene sets were paired against 155 immune disease signatures. Hierarchical clustering was performed based on the similarity that was calculated by Fisher’s exact test of the overlapping genes for each pair. (**I**) Heatmap shows the clustering of drug target gene sets (*columns*) vs. the disease signatures (*rows*). *A*, *B* lists the diseases that are represented by disease signatures showing similarity with drug target gene sets indicated by *C1* and *C2. Bottom bar chart* indicates the number of linked diseases for each drug target shown in the same order as the *above* heatmap. (**II**) *C1*, *C2* tables list the details of drug targets and their linked diseases for drug target gene set clusters shown in (**I**)

We are able to utilize the immune disease gene signatures to investigate whether the current autoimmune or inflammation related disease drugs or drugs at development are targeting these disease gene signatures. Hence, we built target gene sets with the down-stream genes of those drug targets, and evaluated their overlap with disease gene signatures. The heatmap in Fig. 7I shows the clustering of drug target gene sets vs. disease gene signatures. Both drug target gene set cluster C1 and C2 significantly overlap with disease signature cluster A, which mainly represents diseases of psoriasis, dermatitis, IBD, arthritis, and lupus. This is in agreement with the disease indications for most drugs in both C1 and C2 lists (Fig. 7II). However, drug target gene set, cluster C1, also shows significant overlap with the disease signature cluster B, which are enriched with asthma gene signatures. To ensure the validity of our gene signatures, we plotted the targets, which are either approved drugs or drugs under development, with their linked diseases. Drugs linked with more diseases also had more significant overlap with different disease signatures (Fig. 7I), suggesting that our gene signatures represent the gene structure of the disease. For drug target gene sets that are less significantly overlapped with disease signatures, most of them distributed at the left quarter side of the heatmap, their drugs are most likely linked with fewer diseases, and the majority of them are specifically targeting MS. In the bottom bar showing the number of disease indications associated with each target gene, annexin A1, associated with glucocorticoid’s downstream pathway, has the highest number of linked diseases. This gene may play a general role towards the function of these diseases through this steroid pathway; however, we did not find it useful in identifying specific immunological disease manifestations.

**Fig. 8:**
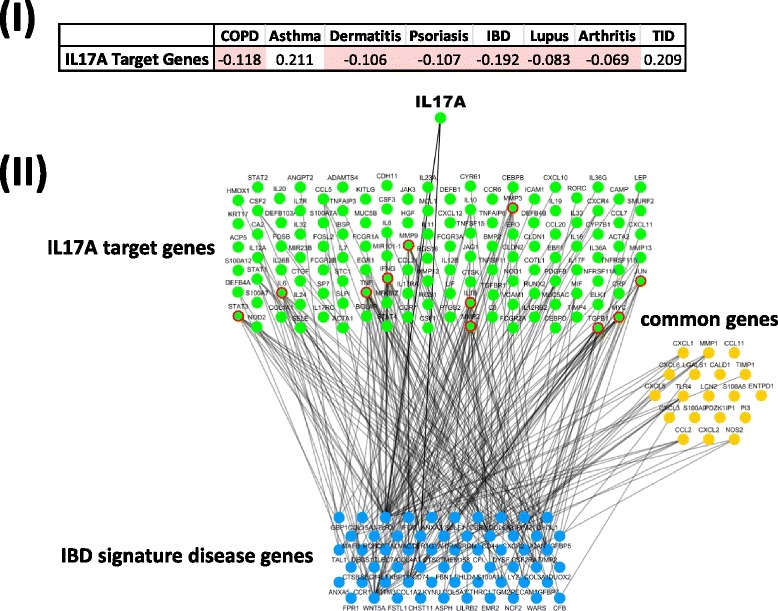
Topological localization of drug target genes and signature disease genes. (**I**) Network-based separation scores of IL17A target genes and signature disease genes of different disease categories. (**II**) Network connection of IL17A target genes and IBD signature disease genes. *Line* represents the direct link between the two groups. Green color: IL17A target gene; *blue color*: IBD signature disease gene; *orange color*: common gene. *Green color* with *red outline*: target genes have direct connection with more than six signature disease genes

In addition to evaluating the direct gene overlap between drug target and disease signatures, we can also assess the topological distribution of drug target genes and signature disease genes on the interactome. As an example, we calculated the network-based separation score of IL17A target genes and signature disease genes of different disease categories. IL17A plays a pivotal role in psoriasis pathogenesis, and its antagonists show great efficacy in moderate-severe psoriasis patients. Shown in Fig. 8I, the negative separation score indicates that IL17A target genes and psoriasis signature disease genes share overlapping ‘neighborhoods’ and are positioned closely on the interactome. In addition to psoriasis, IL17A target genes also show close relatedness with signature disease genes of other indications, such as IBD, COPD, dermatitis, lupus, and arthritis. To explore the connection between IL17A target genes and IBD signature disease genes, the pair with the lowest separation score, we depict the direct links between the two gene sets on the interactome (Fig. 8II). A total of 148 IL17A target genes and 79 IBD signature disease genes can be mapped to the interactome, including 19 genes in common. Out of the 129 IL17A target-specific genes, 87 have a direct connection with at least one signature disease gene of IBD. Among these, 11 genes (circled in red) have connections with more than six signature disease genes. The close connection of target genes and signature disease genes suggests that the alteration of target genes with drug intervention may potentially have a direct impact on those signature disease genes.

## Discussion

In this study, we utilize specifically expressed gene sets to represent the signatures for immune cell subsets, immune related diseases, as well as downstream genes for drug targets. For those gene signatures, we would like to capture the most relevant gene sets without introducing too much noise. For immune cell subsets, we focus on those genes with high specificity scores that rank in the top two percentile. For immune disease signatures, we require genes show significant up-regulation in disease vs. control samples. For a gene set with more than 500 genes, the top 500 genes (~2 % of the genome) with the most significant fold change were selected. In order to evaluate the validity of different cutoffs, we built two additional sets of immune disease signatures by selecting the top 250 genes and 50 genes. Unsupervised hierarchical clustering analyses of immune disease signatures show that the main patterns of co-clustering of different disease categories retain. However, by focusing on a smaller number of gene sets, some of the gene sets fall off from the cluster which may imply that they lose the disease signature (Additional file 1: Figures S2 and S4).

It is critical to understand the role of immune cell subsets in a given disease. The knowledge will help investigators derive relevant cellular models with focused functional studies [9]. However, it’s a daunting task to manage the number of immune cell types with their diverse cellular states and dynamic scale of immune responses [1]. Due to the resource constraints, it’s also not always feasible to sort the interested cell population and profile their transcripts [2]. We applied a computational method to estimate the enrichment of cell specific gene signatures in mixed tissue, with the goal to characterize the recruitment or activation of immune cells in the disease tissues. The similarity matrix along with the unsupervised hierarchical clustering analysis suggests that those specifically expressed gene sets do carry lineage specific signatures, and they retain between human and mouse. However, caution should be used when we interpret the enrichment of immune cell signatures in disease. Depending on the percentage of immune cell subsets in the tissue, the signal can only be captured if the cell type signature is strong enough. Because of this, attention needs to be paid to the positive result. Further validation is required to determine whether the positive result truly came from cell type specific signal. The negative result does not necessarily suggest the missing cell type. It may simply be due to the low percentage of cell type in the whole mixed tissue. It is also worth noting that the cell type gene signatures were constructed based on the expression specificity score [9]. This method identifies upregulated cell type specific, but not necessary uniquely single cell expressed genes in each cell type. Therefore, a gene may show in multiple cell types, such as CD74, present in both DC and B cell, and CD244, present in both NK cell and monocyte (Table 1).

Some common genetic risk factors were reported to be shared in different autoimmune diseases [14]. Pleiotropic module was found to be associated with a wide variety of immune mediated diseases [15]. Understanding the common disease mechanistic basis will help us to identify druggable targets for a broader indication. Systematic analysis of the relatedness of disease gene signatures will shed light on the shared pathways across different diseases.

Autoimmune and inflammatory diseases can be either systematic or organ-specific. Psoriasis is an organ-specific autoimmune disease and it is a chronic inflammatory condition characterized by hyperproliferation of keratinocytes, dermal infiltration of activated CD4+ T cells, and lesional production of proinflammatory cytokines [16]. Atopic dermatitis is an idiosyncratic cell-mediated immunologic acute or chronic reaction to an environmental allergen that comes in contact with the skin [17]. It is an organ-specific manifestation of a systemic disorder [18]. All gene signatures representing both psoriasis and dermatitis were from skin tissues, and they demonstrate high similarities between each other.

Intriguingly, gene signatures from COPD and asthma, two diseases mainly involving lung tissue, do not share high similarity with each other despite being from similar tissue sources. COPD is characterized by airflow limitation that is not fully reversible [19]. All signatures were generated from lung related tissues. Asthma is characterized by variable airflow obstruction, airway inflammation and hyper responsiveness [20]. The majority of the asthma signatures were generated from bronchial brushings or lung biopsies, some from blood, and few from asthmatic chronic rhinosinusitis nasal mucosa. Nevertheless, COPD signatures share limited similarities with signatures from skin (psoriasis/dermatitis), and some other tissues.

Most signatures for sclerosis and T1D are from blood samples even though their diseases are organ-specific. All sclerosis signatures except one are from multiple sclerosis. Multiple sclerosis is a chronic autoimmune disease characterized by demyelination of the white matter of central nervous system [21]. However, the signatures were generated from microarray studies that profiled on samples from blood. Diabetes is also an organ-specific autoimmune disease, the corresponding signatures were derived from blood samples; patient sera, plasma transduced PBMC, or cell lines. The signatures of these two diseases do not share similarity across different studies, even within the same disease. We speculate that the disease gene signatures of organ-specific diseases, such as sclerosis and T1D, may not be accurately represented in blood samples.

For the other three diseases, (1) IBD is a group of inflammatory conditions of the gastrointestinal tract. The major forms include Crohn’s disease and ulcerative colitis [22]. All signatures were from intestine related tissues. (2) Lupus ranges from solely skin involvement to systemic disease. Most of its signatures were from blood samples, fewer from skin and kidney tissues. (3) Arthritis is an inflammation of one or more joints of the body. There are more than 100 different forms of arthritis. The signatures were generated mainly from studies of patients with osteoarthritis, rheumatoid arthritis, and juvenile idiopathic arthritis, with samples from either synovial tissues or blood. Most of the signatures from those diseases share similarity with each other, as well as with signatures from psoriasis and dermatitis. It further implies that blood samples from systemic diseases (i.e. lupus and arthritis) are most likely to carry the gene signatures of the disease.

Most of the autoimmune or inflammatory diseases are complex diseases, resulting from a combination of genetic and environmental factors [12]. GWAS have identified susceptible loci which may lead to insight of disease etiology [23]. One approach to prioritize SNPs is to annotate candidate SNPs with desired genomic features, such as eQTL (expression Quantitative Trait Loci), etc. [23]. The present analysis evaluated only the potential linkage of genetic variation with expression change of directly linked genes, which does not show significant correlation. However, the genetic variation can affect other genes that are either located near the affected gene (cis-eQTL) or in the other part of the genome (trans-eQTL) [24]. In addition, there are limitations of our analysis since we only focus on the highest and most significantly regulated genes. For example, IL23R was reported to be associated with psoriasis by several GWAS studies. Despite that it showed up-regulations in several of the studies we analyzed; it was not selected and included in any of the psoriasis gene signatures due to our selection criteria. The current analysis missed genes with moderate transcriptional level changes. An alternative approach, such as performing the analysis with all regulated genes, could reflect the real eQTL present.

The mapping of drug target gene set vs. disease signature has revealed not only the closeness of different drugs in terms of their targeting disease, but also the potential additional indications. For example, drug target gene sets in Fig. 7IIA and B show high similarity with disease signatures from IBD, psoriasis, dermatitis, arthritis, and lupus, etc. These drugs may have potential indications in those diseases if they have not yet been targeted.

Network based analysis of signature disease genes as well as their topological locations with drug target genes provide us with additional insights to their associations based on the interactome. In addition to shared common genes, we found that some diseases are highly associated with each other by showing direct connections of their signature disease genes. Moreover, some signature disease genes are highly connected with other signature disease genes. This suggests they are the potential central nodal points of the diseases. With better understanding and completion of the interactome, network-based topological analysis of genes and signatures will help to delineate the molecular basis of phenotypical similarity or difference of diseases [9], as well as to identify the targetable nodal points of the diseases.

## Conclusions

Gene signatures representing transcriptional signals for immune cell type and disease were built based on transcriptomic profiles. Signatures were mapped against each other to illustrate conservations of cell subsets within the same lineage, and across human and mouse. The disease signature map indicates the heterogeneity of populations within the disease, as well as connectivity across different diseases. Gene signatures were mapped against each other, cell type vs. disease and drug target vs. disease to build bio-maps based on direct overlap and/or network-based connection. These bio-maps provide insight into disease mechanisms to identify potential targets and develop drugs for broader indications.

## Methods

### Cell type gene signature

Two data sets were used for generating the cell type gene signature. The D-MAP compendium consists of 38 distinct human hematopoietic cells (GSE24759) [25] obtained from blood. The Immunological Genome Project (ImmGen) consists of expression profiles of 249 mouse sorted cells obtained from immunological tissues and blood (GSE15907) [26]. For each data set, data was preprocessed, normalized and sorted based on method in Hu et al. [9]. For each cell type, a nonparametric-expression specificity score was generated for each gene; and genes ranked in the top two percentile were collected in a gene set to represent the gene signature for the cell type. Mouse genes were mapped to human homologs based on HomoloGene. Twenty human and 58 mouse cell type gene signatures (Additional file 2: Table S4) were included in the analysis for Fig. 1 based on the selection of Shay’s paper [7] to represent different cell lineages with matching human and mouse cell types. Detailed gene set information can be found in Additional file 2: Table S1.

### Disease gene signature

One hundred fifty-five gene signatures were generated from 87 studies that involve nine different immune diseases. Disease samples were compared to normal controls. Disease gene signatures were constructed by DEG (Differentially Expressed Genes) with FDR < = 0.05. If there were less than 10 genes, *P* value < = 0.05, and fold change > = 2 were used as cutoffs to choose DEG. For gene sets with more than 500 genes, the top 500 genes with the most significant fold change were selected. Detailed gene signature information can be found in Additional file 2: Table S2.

### Immune drug target gene sets

Drugs with disease indication in autoimmune diseases or diseases involving inflammation were retrieved from Metabase (Thompson Reuters). Those drugs are either FDA approved, or in preclinical or clinical trials. Gene sets were generated by retrieving the direct down-stream genes of the drug target, and those with more than 10 genes were retained. Total 126 drug target gene sets were collected. Detailed module information can be found in Additional file 2: Table S3.

### Network topology of disease genes

The human interactome was downloaded from Metabase (Thompson Reuters). It contains 15,186 genes, with 150,069 interactions. Disease genes were mapped to the interactome, and genes with direct links were retained and visualized using Cytoscape.

### Relationship between gene sets

Fisher’s exact test and Chi-square test were applied to assess the similarity of two gene sets by evaluating the significance of the overlapping genes. Network-based separation analysis was used to evaluate the separation of two gene sets on the interactome according to the method by Menche et al. [11].

$$ SAB=< dAB>-\frac{<dAA>+<dBB>}{2} $$

Network-based separation SAB of two gene sets A and B is quantified by comparing the mean of the shortest distance <dAA> and <dBB> within the respective gene set, to the mean of the shortest distance <dAB> between two gene sets.

## Additional files

Additional file 1:
**Figure S1**. Hierarchical clustering of immune cell gene signatures from human and mouse. Seventy-eight immune cell gene signatures were paired against each other. Hierarchical clustering was performed based on the *P* value of the Fisher’s exact test. The X-axis color bars represent the cell lineage, and species from which the cell gene signatures were derived. Dotted lines separate the cell type clusters which show the lineage conservation of cell gene signatures between human and mouse. In each cluster, cell gene signature tends to cluster more with gene signatures from the same cell subset and the same species, than groups with those from the other species. HSC: Hematopoietic Stem Cell; GN: Granulocyte; MO: Monocyte. **Figure S2**. Hierarchical clustering of immune disease gene signatures. One-hundred-fifty-five Immune disease gene signatures were paired against each other. Hierarchical clustering was performed based on the *P* value of the Fisher’s exact test. The X-axis color bars represent the disease category. Dotted boxes show the clustering of disease gene signatures from different disease categories. **Figure S3**. Hierarchical clustering of immune cell signatures vs. immune disease signatures. Two-hundred-eighty-seven human and mouse immune cell signatures were paired against 155 immune disease signatures. Hierarchical clustering was performed based on the similarity that was calculated by Fisher’s exact test of the overlapping genes for each pair. The heatmap shows clustering of disease gene signatures (columns) vs. the cell type signatures (rows). **Figure S4**. Hierarchical clustering of immune disease gene signatures with different cutoffs. One-hundred-fifty-five Immune disease gene signatures with cutoff of either 250 genes (I) or 50 genes (II) were paired against each other. Hierarchical clustering was performed based on the *P* value of the Fisher’s exact test. The X-axis color bars represent the disease category. Dotted boxes show the clustering of disease gene signatures from different disease categories. Additional file 2:
**Table S1**. Human and mouse cell type gene signatures. **Table S2**. Immune disease gene signatures. **Table S3**. Immune drug target gene sets. **Table S4**. Cell type gene signatures used in Fig. 1.
