# Supplementary material for: Gene signature-based mapping of immunological systems and diseases
Source: BMC Bioinformatics. 2016 Apr 18;17:171. doi: 10.1186/s12859-016-1012-y (PMC4836068; doi:10.1186/s12859-016-1012-y)
Supplement: Additional file 1: — Figure S1. Hierarchical clustering of immune cell gene signatures from human and mouse. Seventy-eight immune cell gene signatures were paired against each other. Hierarchical clustering was performed based on the P value of the Fisher’s exact test. The X-axis color bars represent the cell lineage, and species from which the cell gene signatures were derived. Dotted lines separate the cell type clusters which show the lineage conservation of cell gene signatures between human and mouse. In each cluster, cell gene signature tends to cluster more with gene signatures from the same cell subset and the same species, than groups with those from the other species. HSC: Hematopoietic Stem Cell; GN: Granulocyte; MO: Monocyte. Figure S2. Hierarchical clustering of immune disease gene signatures. One-hundred-fifty-five Immune disease gene signatures were paired against each other. Hierarchical clustering was performed based on the P value of the Fisher’s exact test. The X-axis color bars represent the disease category. Dotted boxes show the clustering of disease gene signatures from different disease categories. Figure S3. Hierarchical clustering of immune cell signatures vs. immune disease signatures. Two-hundred-eighty-seven human and mouse immune cell signatures were paired against 155 immune disease signatures. Hierarchical clustering was performed based on the similarity that was calculated by Fisher’s exact test of the overlapping genes for each pair. The heatmap shows clustering of disease gene signatures (columns) vs. the cell type signatures (rows). Figure S4. Hierarchical clustering of immune disease gene signatures with different cutoffs. One-hundred-fifty-five Immune disease gene signatures with cutoff of either 250 genes (I) or 50 genes (II) were paired against each other. Hierarchical clustering was performed based on the P value of the Fisher’s exact test. The X-axis color bars represent the disease category. Dotted boxes show the clustering of disease gene sign [file 12859_2016_1012_MOESM1_ESM.ppt]

## Slide 1
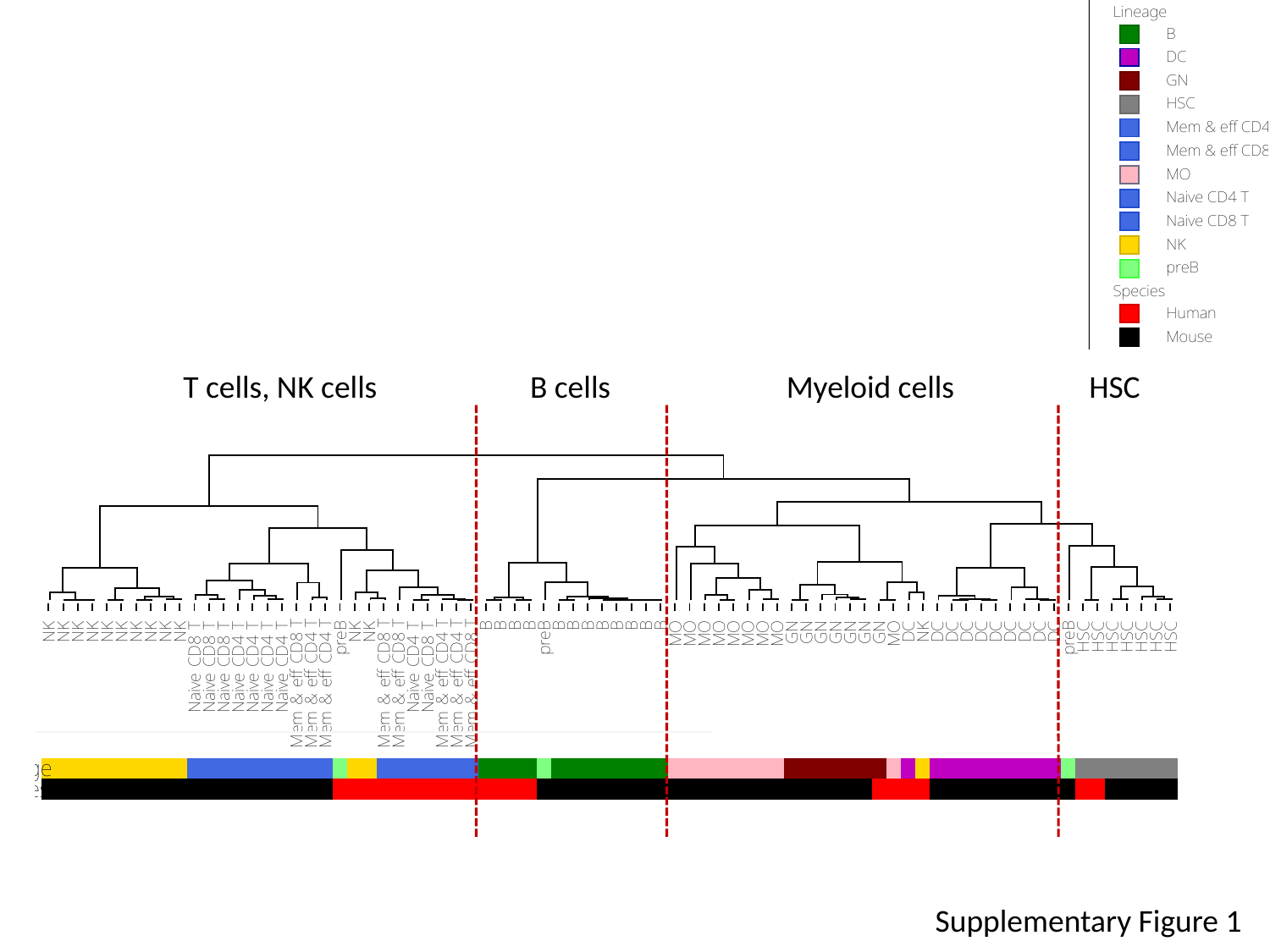

T cells, NK cells
B cells
Myeloid cells
HSC
Supplementary Figure 1

## Slide 2
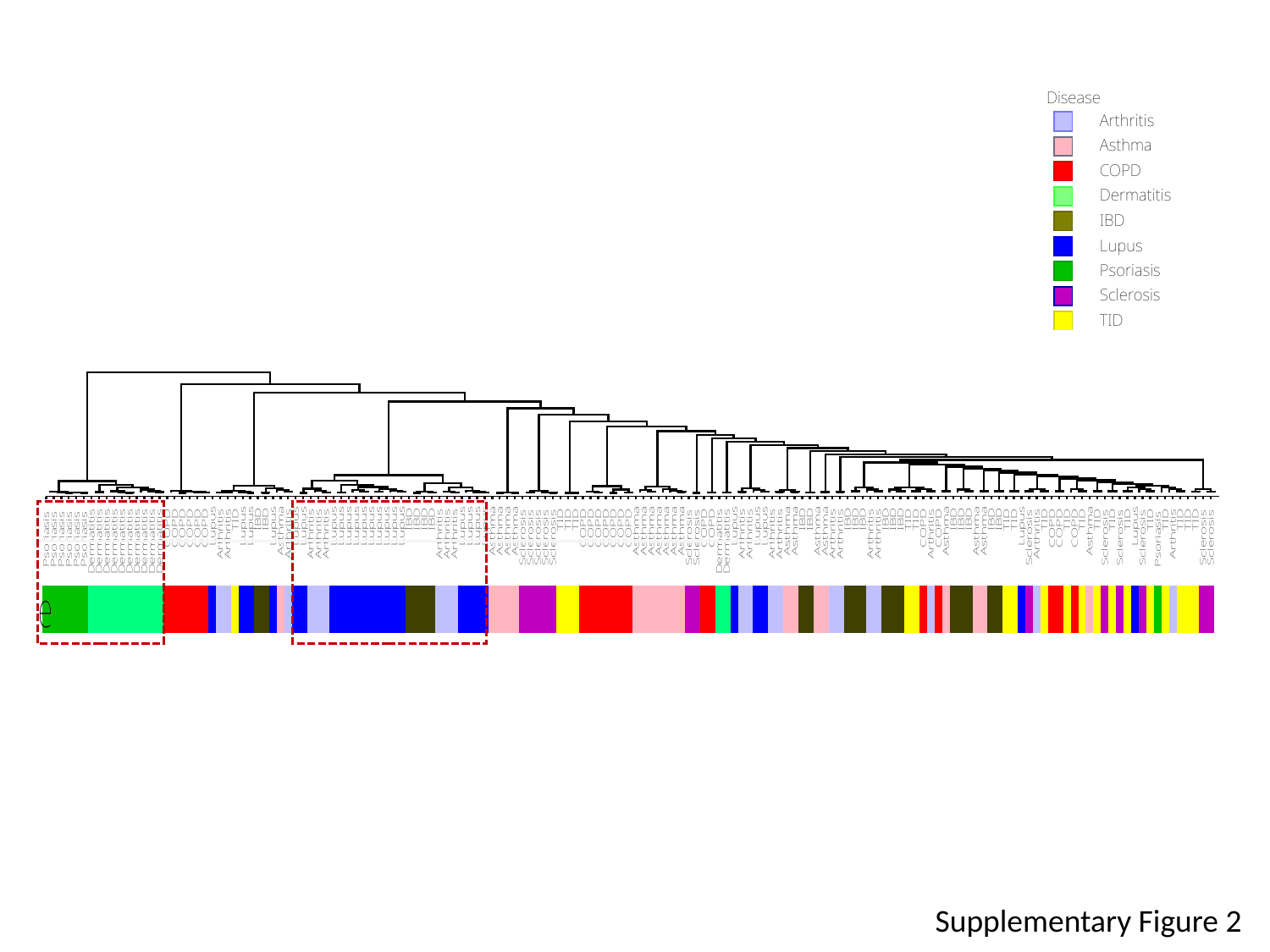

Supplementary Figure 2

## Slide 3
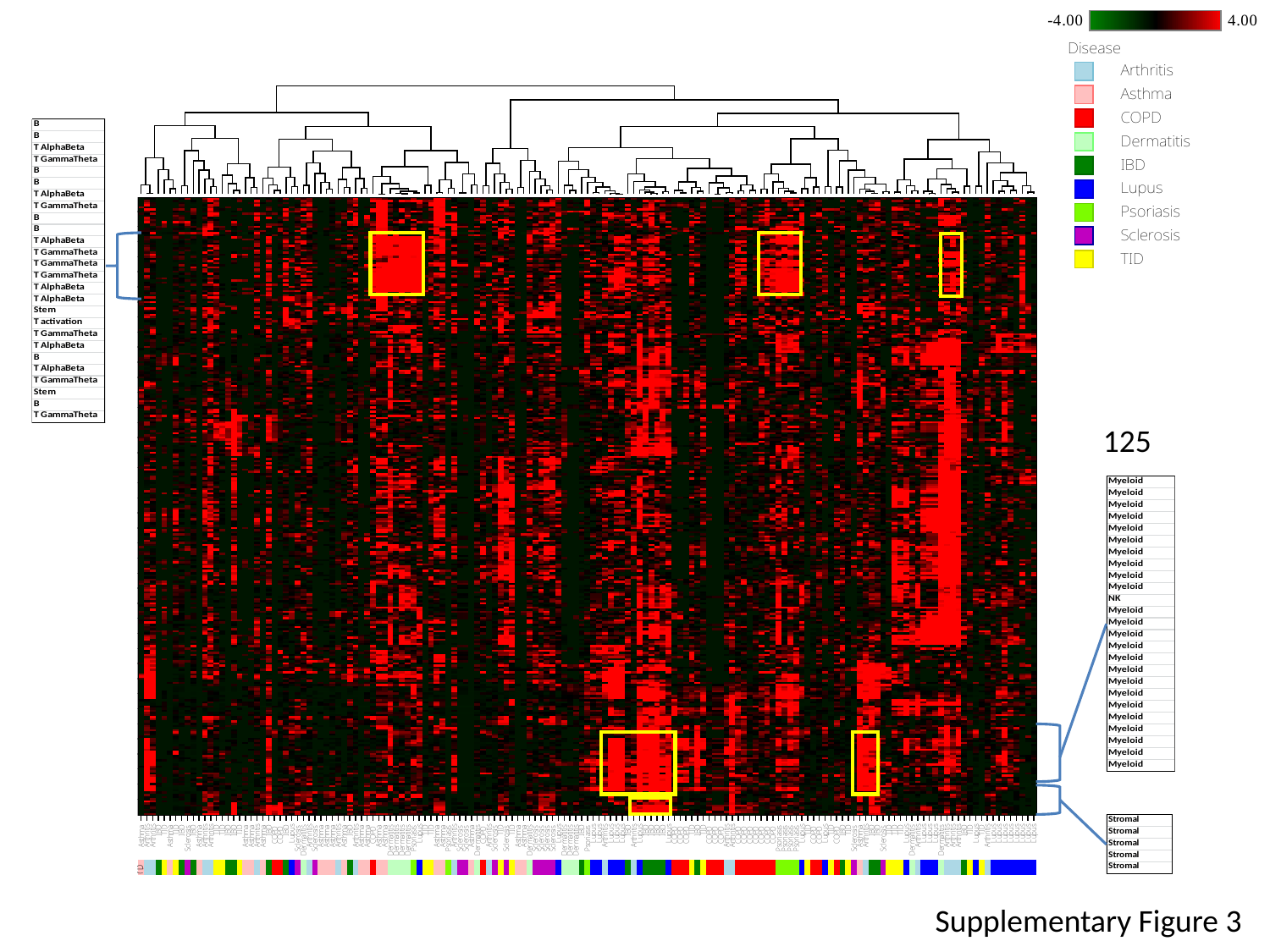

125
Supplementary Figure 3

## Slide 4
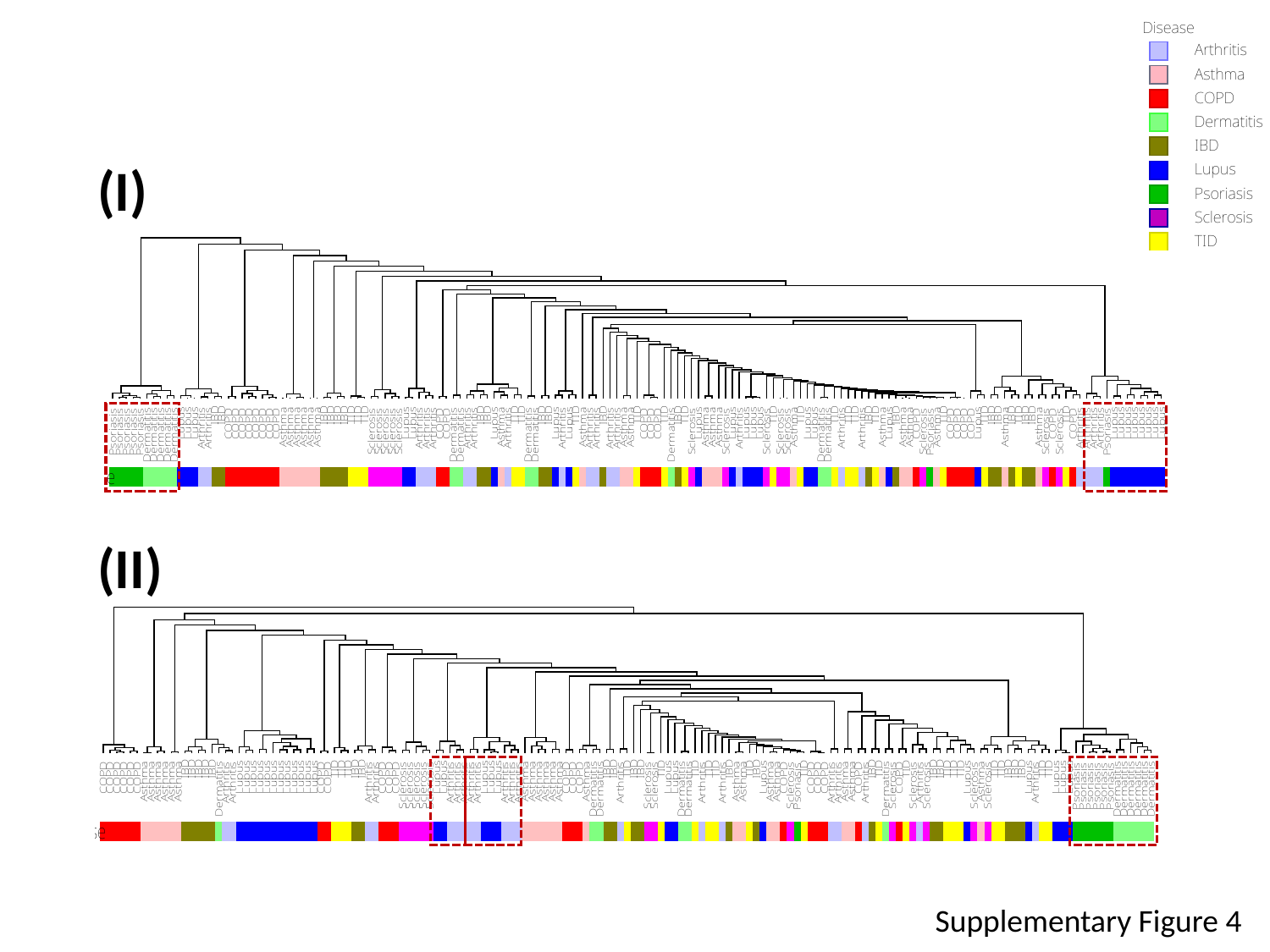

(I)
(II)
Supplementary Figure 4
